# State of the Art and New Directions on Electrospun Lignin/Cellulose Nanofibers for Supercapacitor Application: A Systematic Literature Review

**DOI:** 10.3390/polym12122884

**Published:** 2020-12-01

**Authors:** Abdullahi Abbas Adam, John Ojur Dennis, Yas Al-Hadeethi, E. M. Mkawi, Bashir Abubakar Abdulkadir, Fahad Usman, Yarima Mudassir Hassan, I. A. Wadi, Mustapha Sani

**Affiliations:** 1 Department of Fundamental and Applied Sciences, Universiti Teknologi PETRONAS, Seri Iskandar, Perak 32610, Malaysia; johndennis@utp.edu.my (J.O.D.); abubakarbashir150@gmail.com (B.A.A.); fahatu11@gmail.com (F.U.); mudassir_1800320@utp.edu.my (Y.M.H.); 2 Department of Physics, Al-Qalam University Katsina, Katsina 820252, Nigeria; msani4559@gmail.com; 3 Department of Physics, Faculty of Science, King Abdulaziz University, Jeddah 21589, Saudi Arabia; yalhadeethi@kau.edu.sa (Y.A.-H.); emrzog@kau.edu.sa (E.M.M.); 4 Preparatory Year Deanship, Basic Science Unit, Alkharj 34212, Saudi Arabia; iawadi101@gmail.com; 5 Department of Physics, Faculty of Education, University of Nyala, Nyala 63311, Sudan

**Keywords:** lignin, cellulose, nanofiber, electrospinning, supercapacitor

## Abstract

Supercapacitors are energy storage devices with high power density, rapid charge/discharge rate, and excellent cycle stability. Carbon-based supercapacitors are increasingly attracting attention because of their large surface area and high porosity. Carbon-based materials research has been recently centered on biomass-based materials due to the rising need to maintain a sustainable environment. Cellulose and lignin constitute the major components of lignocellulose biomass. Since they are renewable, sustainable, and readily accessible, lignin and cellulose-based supercapacitors are economically viable and environmentally friendly. This review aims to systematically analyze published research findings on electrospun lignin, cellulose, and lignin/cellulose nanofibers for use as supercapacitor electrode materials. A rigorous scientific approach was employed to screen the eligibility of relevant articles to be included in this study. The research questions and the inclusion criteria were clearly defined. The included articles were used to draw up the research framework and develop coherent taxonomy of literature. Taxonomy of research literature generated from the included articles was classified into review papers, electrospun lignin, cellulose, and lignin/cellulose nanofibers for use as supercapacitor electrode materials. Furthermore, challenges, recommendations, and research directions for future studies were equally discussed extensively. Before this study, no review on electrospun lignin/cellulose nanofiber-based supercapacitors has been reported. Thus, this systematic review will provide a reference for other researchers interested in developing biomass-based supercapacitors as an alternative to conventional supercapacitors based on petroleum products.

## 1. Introduction

Human activities, security, and industrialization depend on energy production and this energy is mostly derived from fossil fuels [1]. However, heavy consumption of fossil fuels poses a serious environmental challenge due to the emission of CO_2_, SO_2_, and NO_x_ into the atmosphere [2]. The World Energy Council (www.worldenergy.org) has estimated that by 2050, the world would demand twice its present energy consumption. Thus, a green approach to an effective energy production system that can replace fossil fuels is necessary [2,3,4]. Recently, flexible supercapacitors are among the most investigated energy storage devices due to their high-power density, rapid charge/discharge rate, excellent cycle stability, and robust nature. The electrochemical double-layer capacitor (EDLC) is a specific type of energy storage system where the capacitance is taken from electrolyte adsorption and alignment at the interface between the electrolyte and a conductive porous electrode with a wide surface area [5]. The efficiency of the EDLC is determined by the properties of its chosen electrode (active) materials. Electrospun carbonaceous materials are used as electrode materials, conducting additives, and supporting substrates in EDLCs due to their excellent electrical conductivity, large surface area, high porosity, mechanical flexibility, and chemical stability [6]. Due to its wide surface area and high porosity, carbon-based supercapacitors draw more and more interest. Recently, carbon-based materials research focuses on biomass materials because of the growing need for sustainable environmental protection. Owing to its high melting point, rich carbon content, and quick speed in pyrolysis, polyacrylonitrile (PAN) is the most suitable precursor for manufacturing high-performance carbon fibers (CFs) as compared to other common precursors (such as polyaniline, pitch, rayon, etc.). PAN precursor electrospinning accompanied by stabilization and carbonization can be used for producing carbon nanofibers (CNFs) having finer and more regulated fiber diameters [7]. However, PAN and other petroleum-based polymers are quite expensive [4]. Additionally, PAN, polybenzimidazole, or pitch, as carbon sources release toxic substances during carbonization [8]. It is therefore necessary to opt for greener carbon sources for the production of CNFs. 

Biomass cellulose (mostly from cellulose acetate, ethyl cellulose, methyl cellulose, etc.), being the most abundant biological macromolecules in nature, is a good precursor material for electrospinning CNFs due to its good flexibility and essential properties [9]. In the last few decades, CNFs has been fascinating, due to its abundance and low cost, while at the same time having special characteristics such as large surface area and high carbon content [10]. Cellulose consists of carbon, hydrogen, and oxygen components, and its carbonization mainly releases carbon dioxide and water. Cellulose does not have an apparent melting point and after successful carbonization, it preserves its physical morphology [8]. Compared to cellulose, it is easier to electrospin cellulose acetate into nanofibers and films because the solubility of cellulose acetate depends on the degree to which the acetate group is replaced [11]. However, cellulose is a macromolecular polysaccharide, a large number of oxygen atoms in cellulose structure are highly autoxidized at high temperatures, thereby causing thermal instability. This thermal instability leads to morphology collapse during carbonization [9]. The second most abundant bio-based polymer is lignin. It is obtained in large quantities from the pulp, paper, and bio-ethanol industries as a coproduct [12]. Lignin is a rich carbon source due to its many aromatic subunits [13]. Electrospun lignin-based CNFs have large surface area, excellent graphitic and porous structures, and superior chemical stability against corrosive elements [14]. However, the structural units in lignin are non-linear and are randomly interconnected by numerous C-C and ether bonds, which together help to make the lignin polymer poorly flexible [15]. Thus, clean lignin materials are comparatively delicate and cannot be easily manipulated. Furthermore, the randomly distributed aliphatic chains separating the aromatic units in lignin reduces its tensile strength and moduli dramatically during thermal treatment [16]. Diversity of lignin biopolymers is another serious problem affecting batch reproducibility of lignin derived CNFs [16]. Nevertheless, lignin may be mixed with plasticizers in order to obtain good fibers [13]. 

The study into CNFs made from a lignin/cellulose blend is a new research field that needs to be explored due to the rising need for energy storage devices based on biomass CNFs. As far as we know, only a few papers on electrospun lignin/cellulose nanofibers for energy storage have been published. Interestingly, the few studies show that supercapacitors based on lignin/cellulose may be an exotic area of research interest in the field of supercapacitors based on carbon biomass precursors. Besides, both lignin and cellulose are green and sustainable, and their extraction methods are very simple, economical, and eco-friendly. The need to investigate this new field is, therefore, very evident. Many reviews on electrospun lignin and cellulose nanofibers have been published. However, very few systemic reports are available on both lignin and cellulose nanofibers. In particular, no review of electrospun nanofibers from a blend of these abundant biomass materials has been published. 

This study is a systematic review of all the literature (available on Web of Science, Scopus, and ScienceDirect) published on the electrospun lignin, cellulose, and lignin/cellulose nanofibers for supercapacitor application between 2010 and 2020. The study reviewed related papers systematically, discussed the advancement of research on the topic, defined areas of research gap, made suggestions, and presented research directions for future studies on the field of carbon-based supercapacitor electrodes. To create a sound base for our study, this systematic review reports on electrospun lignin and cellulose nanofibers independently followed by electrospun lignin/cellulose nanofibers. The papers used in this study are only restricted to supercapacitors application involving lignin and cellulose nanofibers. Thus, this systematic review is likely to inspire researchers working in this field to focus more on biomass-based CNFs as an ideal alternative to petroleum-based CNFs as supercapacitor electrode materials.

## 2. Systematic Review Methodology 

On a general note, a literature review helps to identify and bridge research gaps in a particular research area. A systematic review of literature is quite different from the general review because its protocol is well defined, scientific, and rigorous. The scientific approach employed in systematic review ensures rigorous identification of the research area, summarizes the state of the art research progress, identifies research gaps, and suggests directions for further research [17]. Thus, a systematic review ensures that systematic errors are minimized and improves the legitimacy of data analysis [18]. In effect, this paper is carefully designed to report the emerging trends on supercapacitors derived from lignin and cellulose nanofibers prepared via electrospinning technique. 

### 2.1. Research Questions

With the growing demand for low-cost and high-performance energy storage systems, biomass lignin and cellulose are the preferred materials for future supercapacitors due to their natural abundance, thermal and chemical stability, low cost, durability, and large surface area. As the market for energy storage devices derived from renewable materials continues to grow, many researchers have dedicated themselves to biomass-derived materials because of their economic and environmental advantages. For this reason, cellulose and lignin are the most suitable precursors for biomass-based supercapacitors. Supercapacitors fabricated from electrospun lignin, cellulose, and a blend of lignin and cellulose are discussed as emerging research trends in carbonaceous materials for EDLC application. Thus, the principal purpose of this systematic review is to analyze the state-of-the-art studies on electrospun lignin/cellulose nanofibers for use in supercapacitors. Two research questions are formulated to guide this research in achieving the stated goals.

RQ1: What are the physiochemical properties of electrospun lignin and cellulose nanofibers reported by researchers and what are the electrochemical performance of the fabricated supercapacitors? 

RQ2: How successful are nanofibers derived from electrospun lignin and cellulose blend when used as supercapacitor electrode materials?

The stated research questions will assist in analyzing the research progress on electrospun lignin and cellulose nanofibers as electrode materials for supercapacitors. Since lignin and cellulose are cheap, nontoxic, and readily available, electrospinning of lignin/cellulose precursor into nanofibers with optimized performance could be the next generation supercapacitor material.

### 2.2. Search Strategy

Search strategy for articles relevant to the scope of the research was done in three stages: (1) selecting databases and other sources of data, (2) identifying the keywords to search on databases, and (3) defining the search process used to obtain relevant documents.

#### 2.2.1. Electronic Databases

Three databases were used as sources of research articles for this study: Web of Science, Scopus, and ScienceDirect. These databases are among the most inclusive scientific databases which provide extensive peer review research literature in the field of science. They provide search tools that can be effectively used to obtain bibliographic information as well as research articles. 

##### Keywords Search

To obtain relevant articles, different keywords were used to search databases. The authors noticed that the selected keywords are the most common keywords used in relevant articles. Advanced search by title was done on each database. To enrich our information, a query search was done using different combinations of the keywords as presented in Table 1. 

##### Search Process

Query search was performed for all relevant articles on 11 September 2020. Each query search was performed three times to ensure consistency in the query output. After every query search at different instances, all databases resulted in a fixed number of articles for each keyword except when keywords were changed. For example, step 7 was delimitated as TI = (“electrospinning” AND “nanofiber” AND “lignin”) and TITLE (“electrospinning” AND “nanofiber” AND “lignin”) AND PUBYEAR > 2008 AND PUBYEAR < 2021 on Web of Science and Scopus databases, respectively. The final query search resulted in 11,450 relevant articles from all the databases used (Web of Science = 75, Scopus = 289 and Science Direct = 11,086). Relevant articles used for this systematic review include original research journals, review papers, conference proceedings, and book chapters.

##### Search Criteria

A lot of comprehensive work (experimental and review) on electrospun cellulose and lignin nanofibers have been published. However, our established goals restrict our work to electrospun lignin and cellulose nanofibers for use in supercapacitors only. Therefore, our targeted research articles are those that meet our selection criteria only. From our query search, no literature relevant to the scope of this study was published before 2010. Hence, all articles published before 2010 were not included in the review. Furthermore, the only peer-reviewed articles published in the English language are used for this systematic review. Other criteria—such as journal ranking, publishing country, article access type, etc.—were not included in our selection criteria. 

##### Inclusion and Exclusion Criteria

Criteria for including articles in this systematic review are similar to those reported in a previous study [17]. The inclusion criteria for this study are summarized in Table 2. 

All articles that do not meet the above inclusion criteria were excluded. Articles with no clear contribution to the scope of this systematic review were also excluded during the screening process.

##### Eligibility and Screening of Included Articles

**Figure 1:**
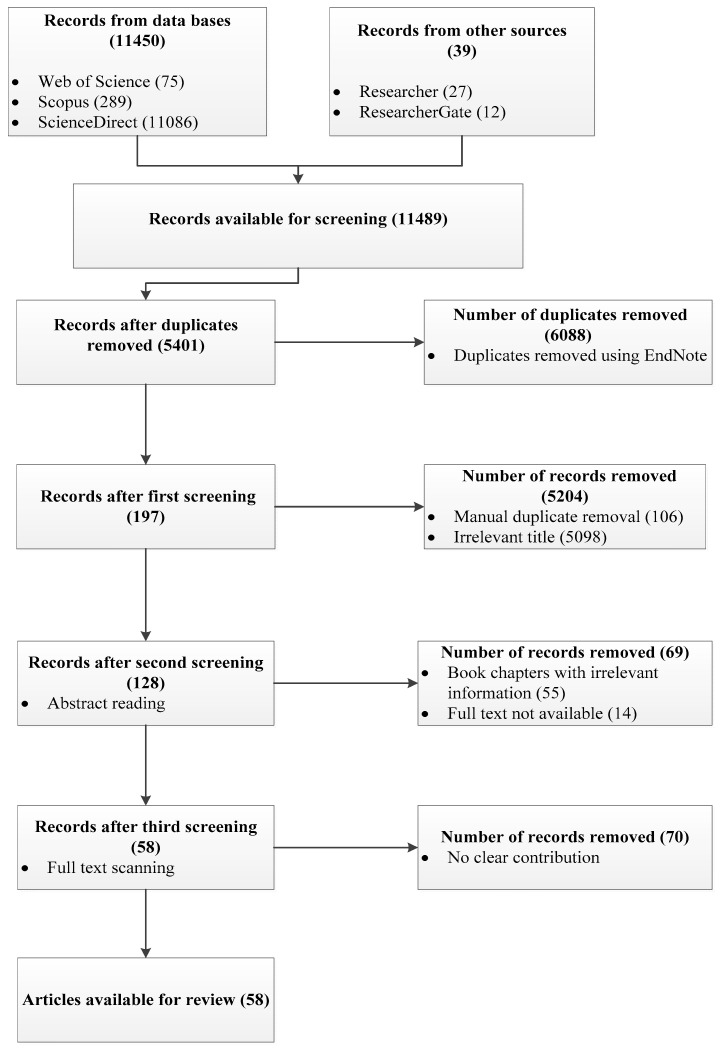
PRISMA flow chart with some modifications [19].

Screening of relevant articles to be included in this systematic review follows the PRISMA (Preferred Reporting Items for Systematic Review and Meta-Analysis) framework [19]. As previously seen in Table 1, 11,450 articles resulted from the query search of the three databases used. Additional 39 articles were obtained from other sources (Researcher = 27 and ResearchGate = 12). Therefore, a total of 11,489 articles were initially used for the screening of most relevant articles. Figure 1 presents the PRISMA framework (with little modification) for the eligibility and screening of included articles.

##### Final Search 

In a systematic review, a final search was done to obtain articles with the most relevant information. As stated earlier, 11,450 references were initially imported to EndNote from databases. Authors equally included 39 articles from other sources. Using EndNote reference manager, 6088 duplicates were detected and removed. However, in the course of manual screening, 106 additional duplicates were further removed. Eligibility and screening processes were done in three stages to obtain articles that strictly fall within the objectives of this study and met the inclusion criteria. The first screening was done by simply reading the titles of the articles. By so doing, 5098 articles were screened out resulting in 197 articles left. These are articles that reported on either; electrospun lignin and cellulose nanofibers for other applications (e.g., tissue engineering, water and wastewater treatment, heavy metal adsorption, lithium-ion batteries and fuel cells, sensors, etc.) or lignin and cellulose nanofibers for supercapacitor applications prepared via other methods (e.g., hydrothermal, pyrolysis, template synthesis, etc.).

To further scrutinize our remaining literature, a second screening was performed by reading the abstracts of the articles that passed the first screening. Out of 197 articles, 14 articles were screened out because we could not obtain the full articles. Also, 55 book chapters with no significant information were screened out. At the end of this screening, 128 articles were obtained. The final screening was performed by scanning through the entire articles remaining, to further determine their relevance to the scope of this study. The authors observed that 70 articles have no clear contributions to offer to the research topic and were therefore screened out. Most of these articles were conference papers that did not express a concise methodology or did not present a sufficient explanation of results to answer our research questions. 

##### Publication Bias

Many biases are likely to arise when looking for the right research for writing the systematic review. Bias involving arbitrary search constraints such as geographic locations, search items, electronic databases, publishing languages or publication period may be included by the review authors. Bias is a methodological mistake in research, leading to the adoption of findings and conclusions without taking adequate account of the probability of inaccurate or misleading reports. Publication bias exists where the findings of published research vary from the results of unpublished studies. We may assess publication bias by comparing the findings of published and unpublished research addressing the same research question [20,21].

**Figure 2:**
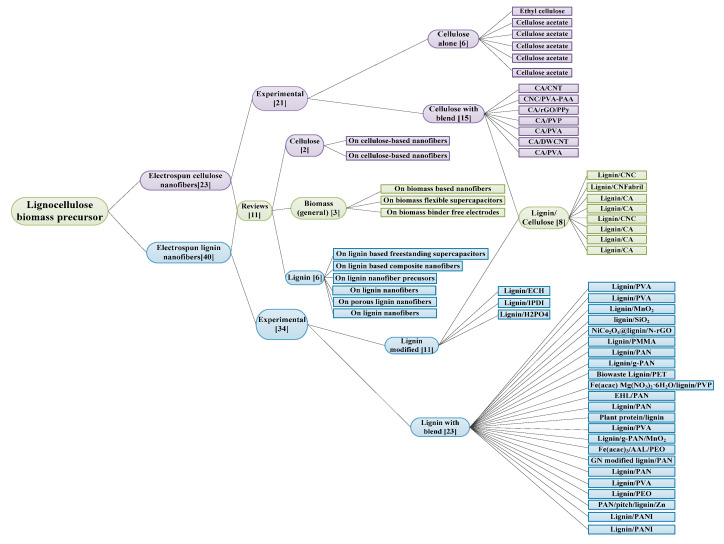
Taxonomy of research literature on electrospun lignin and cellulose nanofibers.

Bias analyses are relevant because they allow the authors and the readers to assess how accurate and reliable the data included in the study are. For this study, publication bias was not estimated because authors were neither able to get unpublished research work on the topic nor published articles with negative/irrelevant results which would be compared with the published research work with positive outcomes. At the end of the screening of the included articles for eligibility, a total of 58 relevant articles with a clear contribution to the study were eventually collected for the systematic review at the end of the eligibility and screening process. These articles were further studied extensively to establish a full framework of literature taxonomy and to map out our research. The development of a taxonomy of literature from our included articles was based on three categories of literature: articles on electrospun lignin nanofibers, articles on cellulose nanofibers, and articles on electrospun lignin/cellulose nanofibers. The distribution of articles used in the literature taxonomy is shown in Figure 2. In the process of developing a research framework for this systematic review, 9 additional articles (although out of scope) were included in the study because of contributory information obtained from them. Therefore, 67 references were used for this study to map out our research.

## 3. Results and Discussion

### 3.1. Descriptive Analysis of Included Articles

**Figure 3:**
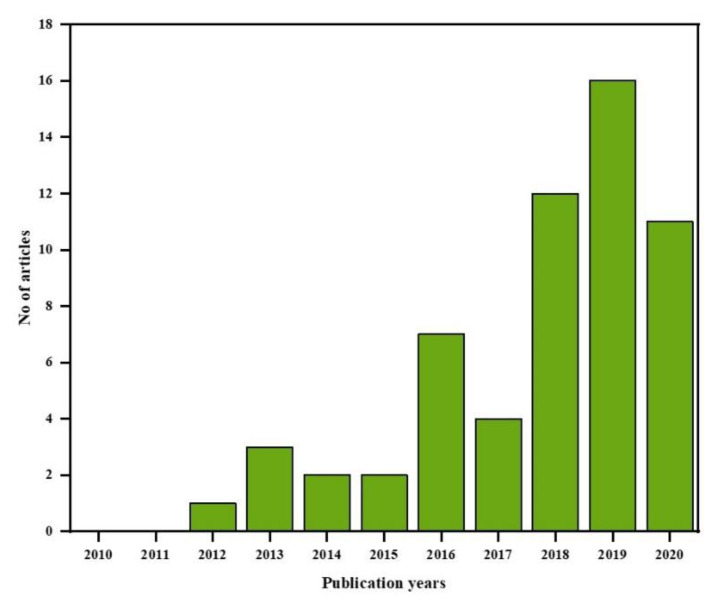
Distribution of included articles on electrospun lignin and cellulose nanofibers as electrode material for supercapacitor based on year of publication.

While research into supercapacitors based on lignin/cellulose is currently at its early stage, it is important to review this new field of study to improve the research community’s awareness of the subject. It is expected that this review will help to divert the attention of researchers from petroleum-derived carbonaceous electrode materials such as PAN, rayon, and pitch to renewable and sustainable biomass-based electrodes. If explored, this field of study will offer sufficient opportunity to study lignocellulose-based electrode materials. The evolution of research articles included in this study per years of publication is presented in Figure 3. 

**Figure 4:**
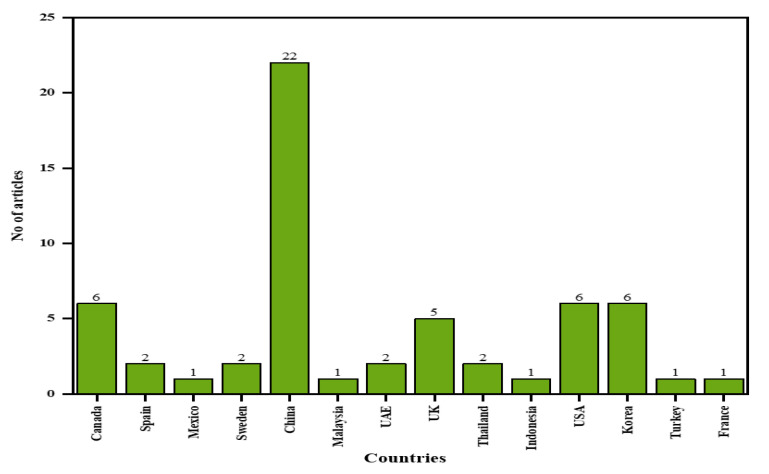
Distribution of included research articles on electrospun lignin and cellulose nanofibers as electrode material for supercapacitor based on countries.

Comparatively, the distribution of research articles used in this study according to countries of authors is shown in Figure 4. From the figure, China has the highest number of published articles (22) on this topic. Other countries with a considerable number of publications in the area include Canada, the USA, and Korea with six published articles each.

**Figure 5:**
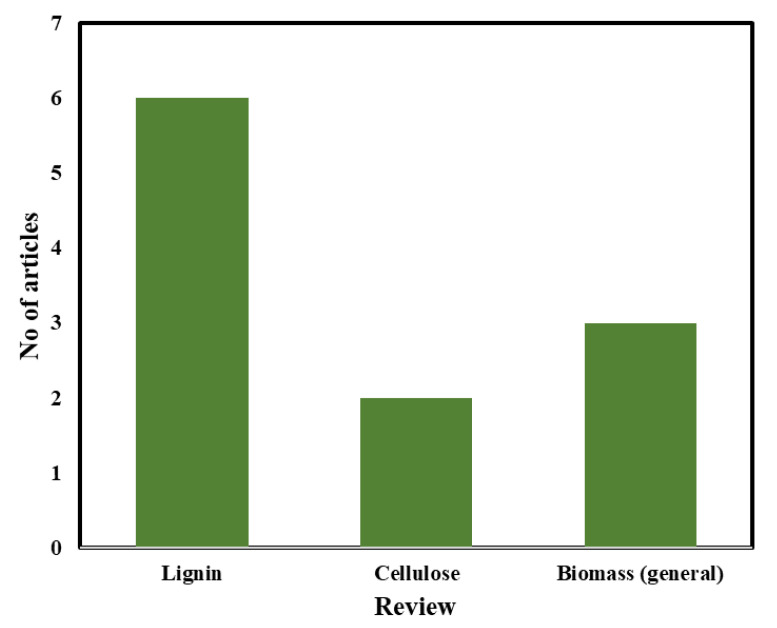
Distribution of review articles on electrospun lignin, cellulose, and biomass-derived nanofibers as electrode material for supercapacitor included in articles of present review.

Several review articles on electrospun lignin and cellulose nanofibers were published over the years. For this study, these reviews are divided into three categories: (1) reviews on electrospun cellulose nanofibers for supercapacitors, (2) reviews on electrospun lignin nanofibers for supercapacitors, and (3) reviews on biomass-derived nanofibers for supercapacitors. In this regard, only the review papers that are highly relevant to our study and fulfil our inclusion criteria have been selected. After the examination of the available review publications, 11 review papers (19% of total included articles) with clear relevance to our goals were included in this systematic review. The number of the review articles classified according to their categories (as shown in Figure 5) are as follows: six review articles investigated electrospun lignin nanofibers, two investigated electrospun cellulose nanofibers, and the remaining three investigated general biomass-based carbon nanofibers. 

**Figure 6:**
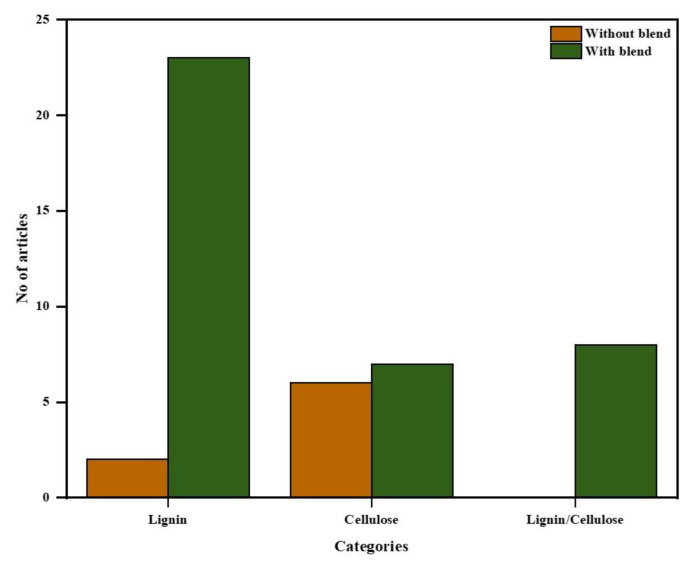
Distribution of included experimental research articles according to the category of electrode materials on lignin, cellulose, and lignin/cellulose nanofibers.

Experimental research articles cover 81% (47 out of 58) of the total relevant articles used in this study to create literature taxonomy to develop the research framework. The distribution of the included experimental research articles based on electrode active materials (lignin, cellulose, and lignin/cellulose nanofibers) are shown in Figure 6.

**Figure 7:**
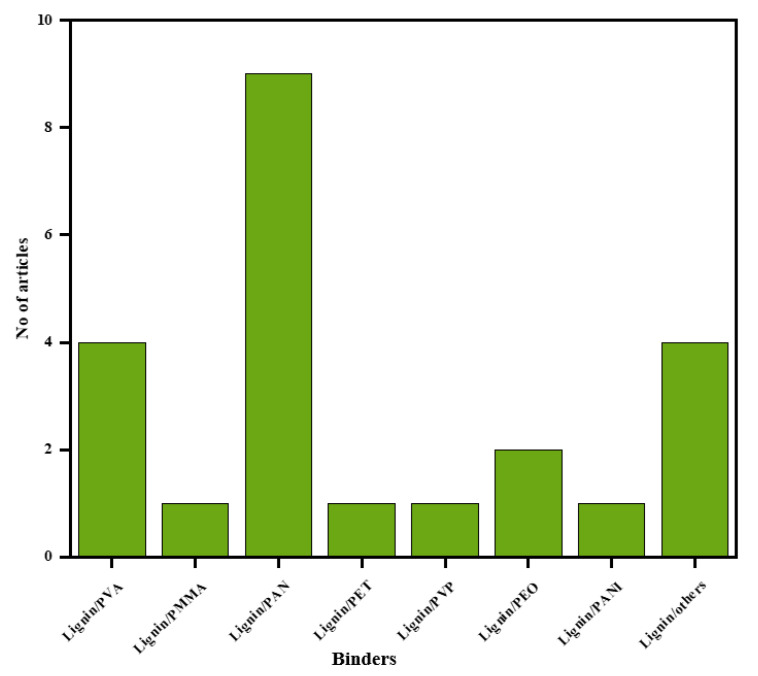
Different binders blended with lignin to form lignin-based nanofibers.

Out of the 47 articles, 21 reported on electrospun cellulose nanofibers, 34 reported on electrospun lignin nanofibers and 8 reported on electrospun lignin/cellulose nanofibers. For electrospun lignin nanofibers, 23 out of 34 reported on various polymeric binders blended with lignin to successfully electrospin lignin precursor into nanofibers. Figure 7 presents the number of articles (used in this study) for different polymeric binders blended with lignin. Furthermore, 5 out of 8 reported articles (62.5%) on electrospun lignin/cellulose nanofibers investigated the performance of lignin/cellulose acetate (CA) blend while 3 (37.5%) investigated the performance of lignin/nanocellulose blend.

### 3.2. Studies on Electrospun Lignin and Cellulose Nanofibers

Based on the included literature, this section discusses the research progress in the field of supercapacitors derived from electrospun lignin and cellulose nanofibers over the years (2010–2020) based on Web of Science, Scopus, and Science Direct electronic databases.

#### 3.2.1. Studies on Electrospun Cellulose Nanofibers

Cellulose is the most abundant natural macromolecule and the main ingredient of lignocellulose biomass. It has a high carbon content of around 44%, high stability, and outstanding porosity due to its hierarchical composition and highly functional rigid linear chains [22,23]. Cellulose is well known as a precursor material for the processing of CNFs. In the late 60 s rayon (a fabric made from cellulose) based fibers became the first commercial carbon fibers [24]. Because cellulose does not dissolve in many organic solvents, cellulose nanofibers are prepared from different derivatives of cellulose such as cellulose acetate (CA) and ethyl cellulose (EC) [8]. In this study, 13 articles on electrospun cellulose nanofibers meet our inclusion criteria. Therefore, this section presents a review of these 13 articles on electrospun cellulose nanofibers.

##### Precursor Solution for Electrospun Cellulose Nanofibers

**Figure 8:**
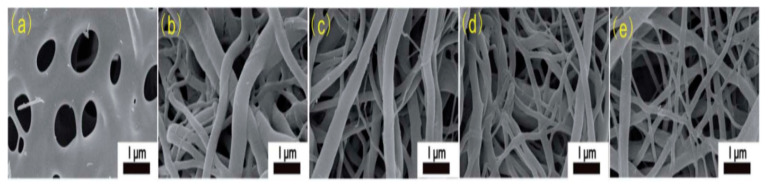
SEM images of electrospun cellulose acetate nanofibers (dissolved in acetone/DMAc) at different ZnCl_2_ content (**a**) CACNF-0, (**b**) CACNF-ZnCl_2_-2%, (**c**) CACNF-ZnCl_2_-5%, (**d**) CACNF-ZnCl_2_-10%, (**e**) CACNF-ZnCl_2_-20% [25].

In CNF formation, the selection of an appropriate solvent is extremely significant. The right solvent that can evaporate quickly during the electrospinning process is required to facilitate jet elongation leading to nanofiber formation. Cellulose electrospinning solution is difficult to prepare because it is normally not soluble in most organic solvents. However, cellulose acetate (CA) is much easier to electrospin because the solubility of CA depends on the degree to which acetate group is replaced. Therefore, CA is the most widely used cellulose derivative to prepare cellulose nanofibers. According to one report, CA was dissolved in acetone and dimethyl acetamide (DMAc) to electrospin CA nanofibers followed by hydrolysis in NaOH/ethanol solution at room temperature for 12 h prior to thermal treatment [25]. These nanofibers were easily electrospun because the mixture of DMAc and ethanol provided ample surface tension to interplay with the viscoelastic force, thereby forming the Taylor cone. As can be seen in SEM images in Figure 8, the resultant nanofibers show a melting phenomenon due to morphological collapse. The morphological collapse could be due to short soaking time in DMAc/ethanol solution leading to incomplete deacetylation. Formation of higher fibrous morphology could be attributed to increasing ZnCl_2_ content in the precursor solution. Ths reveals that ZnCl_2_ increases thermal and structural stability by efficient ion interlinking of CA macromolecules, thus promoting dehydrogenation.

In another study by Y. Lie et al. [26], CA (in DMAc/acetone) were electrospun into cellulose nanofiber. Prepared nanofibers underwent deacetylation using NaOH/ethanol solution (at 25 °C for 24 h) to convert cellulose acetate to cellulose before stabilization. Topographies of the resultant nanofibers show some random glassy interwoven network without noticeable fracture. We can attribute the absence of morphological collapse here to adequate hydrolysis time. Therefore, sufficient hydrolysis time is required to fully deacetyl CA.

Ethyl cellulose (EC) is another cellulose derivative used to electrospin cellulose nanofibers. EC electrospinning precursor solution was prepared by dissolving EC in a mixture of dimethyl formamide (DMF) and acetone [27]. Prepared nanofibers show beads when pure DMF was used as the only solvent. Beads gradually disappear when acetone was introduced increasingly with beads free nanofibers formed at 2:3 DMF/acetone. However, a higher concentration of acetone led to the formation of beads on strings. While the disappearance of beads could be attributed to the many ethoxy groups in ethyl cellulose which are readily soluble in acetone, the formation of beads on string can indicate that surface tension prevailed over electrostatic force during electrospinning. Also, the reoccurrence of beads at higher concentrations of acetone could be due to the rapid evaporation of acetone during the electrospinning process causing nozzle clogging.

The interaction between solvent and polymer (solvent quality) is another factor that significantly affects polymer chains which are responsible for nanofiber formation. CA precursor solution was prepared using dichloromethane (DMC)/ethanol and DMF/ethanol at different concentrations [28]. At a higher amount of CA in DMF/acetone, smooth fibers were obtained. As the amount of CA was reduced, fibrous morphology of the nanofibers was destroyed, leading to beads formation and particles like structures. Loss of fibrous morphology could be explained in terms of surface tension. Viscoelastic force is required to stretch solution droplets to form nanofibers. At low CA amount, the surface tension was not high enough to fully stretch the solution during ejection. Similarly, the spinnability of CA in DCM/acetone was low compared to DFM/acetone. One way to explain this is by considering the solubility parameter of CA, DMF, and DCM (25.1, 24.8, and 20.2, respectively) [29]. Since like dissolves like, we expect CA to dissolve more effectively in DMF than in DCM.

M. S. Nasir et al. [30] incorporated SiO_2_ nanoparticles into cellulose acetate spinning solution in acetone/DMAc to prepare cellulose electrospinning solution. Although the addition of SiO_2_ reduced fiber diameter, spinnability of the precursor solution was not affected. SiO_2_ has been shown by other researchers to improve electrical properties by improving the porosity of prepared CNFs but not the solubility or spinnability of the precursor solutions [31,32,33]. Retention of fiber diameter of the prepared nanofibers after thermal stabilization indicated that SiO_2_ nanoparticles effectively controlled the thermal stability of prepared nanofibers.

##### Electrospinning Technique

The method for electrode materials synthesis plays a crucial role in the regulation of substrate structures and characteristics (presented in Table 3). Some of these methods include, among others; electro-polymerization/electrodeposition, solgel, in-situ polymerization, direct coating, chemical vapor deposition (CVD), electrospinning, vacuum filtration technique, hydrothermal/solvothermal, co-precipitation, template synthesis, etc. [34]. From the above methods, electrospinning is ideal for the manufacture of fine fibers of nanometers range (to a few micrometers). Electrospinning is a flexible technique in nanofabrication based on repulsive electrostatic forces in order to create a viscoelastic solution in the nanofibrous form. While there are many innovations in electrospinning systems, all configurations are based on three simple components: high voltage power supply, a viscoelastic solution distribution mode, and a fiber recovery system [35].

Generally, electrospinning variables can be classified into three parameters: solution characteristics (e.g., viscosity, surface tension, etc.), control variables (e.g., flow rate, electric field, etc.), and environmental parameters (e.g., relative humidity, temperature etc.) [35,39]. When a liquid droplet experiences an electric field, an electrostatic charge is deposited at the tip of this droplet [40]. At sufficiently high pressure, the electrostatic force that is produced by the charged droplets repels the surface tension at the end of the needle; thus forming the Taylor cone, which releases the jet through the spinneret when the coulombic force surmounts the surface tension [41]. The fiber diameter depends on the formation of Taylor’s cone; thus, it is linked to several experimental variables such as the nature of the solvent, the viscosity of the suspension; injection flow, the needle distance, and the voltage applied. Electrospinning increases the process efficiency, allowing fiber of many different sizes to be produced and the process is reproducible as long as these variables are strictly regulated [32,42]. Furthermore, operating the electrospinning machine does not demand a high level of expertise and the technique does not require a vacuum environment to spin the precursor solution into fibers. 

##### Thermal Treatment

**Figure 9:**
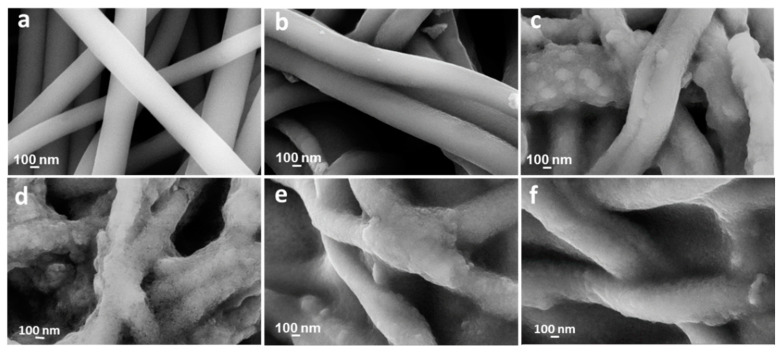
High-resolution SEM images of cellulose acetate nanofibers hydrolyzed in different concentration of NaOH for varying ethanol-water solution. (**a**) CNFs-E, (**b**) CNFs-90/10, (**c**) CNFs-70/30, (**d**) CNFs-50/50, (**e**) CNFs-30/70, and (**f**) CNFs-10/90. Reprinted with permission from ref [44].

Cellulose is insoluble in most organic solvents. However, cellulose derivatives such as CA and methyl cellulose are the common precursors to prepare cellulose nanofibers. Cellulose, for example, does not have an apparent melting point, but CA melts at around 227–277 °C and CA nanofiber loses its fibrous morphology when exposed to high-temperature thermal treatment [43]. Therefore, CA (or other cellulose derivatives) are modified before being subjected to high-temperature treatment. For example, CA was hydrolyzed to convert it to cellulose before thermal treatment. The effect of hydrolysis rate of electrospun cellulose acetate nanofibers in NaOH (at various ethanol/water ratio) was investigated [44]. The resultant nanofibers were thereafter thermally stabilized in air at 240 °C for 1 h and subsequently carbonized at 1000 °C for 2 h. As seen in Figure 9, fine nanofibers were obtained when cellulose acetate was hydrolyzed in NaOH/ethanol. Rough interbedded surfaces indicating the melting of unhydrolyzed CA were observed when NaOH was mixed with ethanol/water. The reason for this could be due to a homogeneous deacetylation reaction when ethanol solution was used. Ethanol easily penetrates throughout the CA matrix whereas water diffuses relatively slowly in the CA matrix.

##### Influence of Fiber Morphology on Supercapacitor Performance

Since the electrode active materials determine the efficiency of the supercapacitor, tunning the right morphology of nanofiber-based materials can greatly influence the energy density of the supercapacitor. The effect of various morphological parameters on the performance of the supercapacitor is presented in this section.

Fiber surface area: For an ideal carbon electrode, a large surface area may provide ample electrode/electrolyte interfaces for ion or charge storage. Supercapacitors energy storing capacity is substantially enhanced with increased real surface area and maximized pore size for delivery of carbon materials without losing electrical conductivity [45]. C. Ma et al. [46] showed that the capacitance retention of PAN/CA nanofibers increased with an increased surface area of the prepared nanofiber. Highest capacitance retention (63% at 10 Ag^−1^) was attributed to the largest surface area (1355 m^2^ g^−1^ for 7:3 PAN/CA ratio).

Porosity: Suitable porosity in materials is necessary for the transport of electrolytes and ion storage. Thus, selected pores in the electrode should combine ion buffering reservoirs (macropores), strong channel transports (mesopores), and ion storage sites (micropores) [47]. J. Kim et al. [33] prepared porous PAN CNFs by etching silica from PAN nanofiber surface. The porous nanofibers showed 38% higher areal capacitance compared to nanofibers without porosity tailored on the surface of the electrode. 

Fiber diameter: Nanofibers with lower fiber diameter have a much higher specific surface area with fewer defects which results in better electrochemical performance. Y. lie et al. [26] showed that glassy CNF carbonized at 1400 °C exhibit the lowest average fiber diameter of 45 nm and the highest electrical conductivity of 93.5 Scm^−2^. Highest electrical conductivity at smaller fiber diameter is due to high surface area and less defects on the fiber structures. 

Disordered fibrous morphology: The ordered arrangement of fibers is another factor that greatly influences the performance of the fabricated supercapacitor. M. Cho et al. [48] reported that interconnected nanofibers allow rapid transfer of electrolyte ions thereby improving the electrochemical performance of the fabricated supercapacitor. In a different study, beaded CA nanofibers were reported by J. Wu et al. [49]. Beaded fiber produced from CA (in acetone/DMAc) recorded a lower conductivity (0.253 µS cm^−1^). Hollow fibrous morphology has also been reported by B. Yu et al. [50] prepared a porous coaxially electrospun lignin nanofiber with a BET surface area of 1200 m^2^ g^−1^. High porosity is attributed to the evaporation of ethanol (core material) during spinning.

#### 3.2.2. Studies on Electrospun Lignin Nanofibers

Lignin is the second most abundant natural bioresource material, after cellulose. It provides structural supports to plants because of its rigid nature. According to the International Lignin Institute, there are approximately 300 billion metric tons of lignin on earth with an annual estimated production of 40 to 50 million tons as by-products of the pulp and paper industries and biorefineries. Lignin has a high carbon content, almost 60–65%, which indicates a possible high yield following carbon fiber processing [51]. Carbon fibers from lignin precursor material were first reported by Kubo et al. [52]. The authors proposed the conversion of lignin into functional polymers by an efficient separation procedure to extract the spinnable material from lignin. After thermal stabilization, the prepared fibers were carbonized to convert lignin fibers to carbon fibers. Currently, electrospun lignin nanofibers are prepared either by physically blending lignin with other polymers or by chemically modifying lignin structure [36,42,53,54,55]. This section will analyze those articles that investigated electrospun lignin nanofibers. 

##### Chemical Modification of Lignin Structure

Foreign elements can be introduced into lignin precursor solution to alter the non-linear and randomly entangled numerous C-C and ether bonds which makes lignin rigid and difficult to electrospin. Using a simple heated single spinneret system, Tunnapat and Surawut [36] were able to electrospin water-soluble lignin nanofiber by adding glycerol into the precursor solution. Addition of glycerol reduced surface tension of the precursor solution thereby improving the spinnability of lignin. At a higher glycerol ratio, the fiber diameter and BET surface area increase while electrical conductivity decreases. Decreased fiber diameter can be due to glycerol evaporation during thermal stabilization whereas decreased electrical conductivity may mean that glycerol plays a major role in altering the lignin structure and improving spinnability. Similarly, increased surface area at higher glycerol ratio can be attributed to successive evaporation of glycerol during thermal stabilization, reducing the resulting average fiber diameter. In another report, lignin/H_3_PO_4_ nanofibers were prepared via coaxial electrospinning technique of Alcell lignin in ethanol (core spinning solution) and pure ethanol (shell spinning solution) [12]. Electrospinning of lignin nanofiber was assisted by the presence of H_3_PO_4_ in the precursor solution. H_3_PO_4_ could effectively dehydrate lignin phenolic-OH group to form phospholipid bonds between lignin molecules, thereby increasing the spinnability of lignin [56]. Lignin nanofibers were also prepared by adding isophorone diisocyanate (IPDI) to the spinning solution [57]. IPDI could link adjacent lignin molecules by establishing stable covalent bonds between them. SEM images (not shown) of lignin nanofibers prepared by either phosphating process or by covalent bonding neither have beads nor show evidence of morphological collapse after carbonization. Evidently, stable lignin fibers were formed because of continuous dehydration of phenolic hydroxyl groups in lignin by H_3_PO_4_ and IPDI, respectively. 

##### Physical Blending of Lignin

Despite natural abundance of lignin and high surface area of lignin nanofibers, their poor electrical properties result in poor electrochemical performance of the lignin-based electrode. To improve the spinnability and morphological properties of lignin nanofiber-based electrode, lignin can be blended with other binding polymers. Similarly, blending lignin with other binders enhances the electrochemical properties of the prepared nanofibers. In this section, the effect of various binders on electrospun lignin nanofibers reported in our included articles are discussed. The processing conditions for reported polymers blended with lignin for application in supercapacitor devices are summarized in Table 4. 

Some of the major polymeric binders reported in Table 4 and their influence on the physiochemical properties of electrospun lignin nanofibers include:

Polyacrylonitrile (PAN): Because of the excellent flexibility and fiber formation ability of PAN, Lignin has been blended with PAN to produce nitrogen-rich nanofibers with good plasticity [69]. X. Wang et al. [62] blended enzymatic hydrolysis lignin (EHL) with PAN at different PAN/EHL ratios (100/0, 60/40, 40/60, and 30/70). Surface area of the prepared nanofiber increased with increasing EHL content in the precursor solution. However, the highest specific capacitance of 305.7 F/g was recorded for 60/40 PAN/EHL. In the same respect, J. H. Park et al. [64] prepared a CNF based on lignin/PAN precursor. The optimum performance was recorded for 50/50 lignin/PAN but not lower or higher ratios. Although the largest surface area with large pore volumes was obtained at a 75/25 lignin/PAN ratio (due to the high porosity of lignin), the specific capacitance of the nanofiber is quite low. Generally, low specific capacitance at lower lignin content can be attributed to low surface area and pore volumes while low specific capacitance at higher lignin content could be attributed to a large amount of either inaccessible micropores or performance-limiting macrospores. 

**Figure 10:**
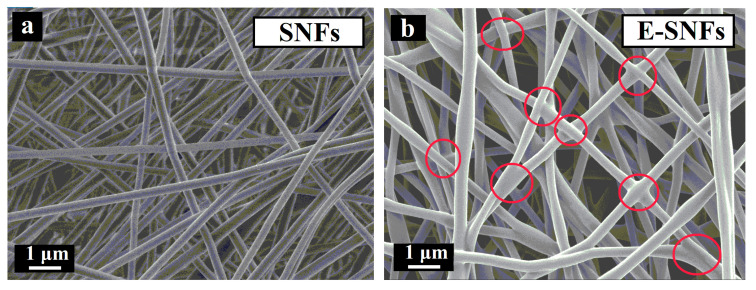
SEM images of (**a**) lignin/PAN nanofibers and (**b**) esterified lignin/PAN nanofibers. Reprinted with permission from ref [71].

In another study, butyric anhydride (BA) was added into lignin/PAN precursor solution prior to electrospinning [71]. While PAN improves the flexibility of the electrospun nanofiber, BA forms ester bonds between hydroxyl groups in lignin anhydride groups in BA. Improved performance of the resultant nanofiber could be attributed to the esterification of lignin which improves inter-fiber bonding (for efficient electron transfer) during carbonization. Figure 10b shows the numerous interfiber bonds formed due to BA esterification of lignin. In a different study, lignin/PAN fiber diameter was reported to increase with increasing lignin concentration. A similar observation was reported by R. Ding et al. [59]. This could mean that butyrated lignin/PAN nanofibers show better thermal and rheological properties compared to lignin/PAN nanofibers. The reason could be due to the fact that esterification of lignin results in higher molecular thermal mobility by reducing intermolecular interactions. On a more general note, all lignin/PAN nanofibers reported in this research identify that blending lignin with PAN improves the mechanical properties of the prepared nanofibers.

Polyvinyl alcohol (PVA): Although nanofibers cannot be fabricated from lignin alone due to its heterogeneity and branching structure, electrospun lignin/PVA nanofibers showed impressive electrochemical properties in addition to interwoven beads free nanofibers. For comparison, pure PVA nanofibers were equally synthesized. Compared to pure PVA nanofibers, lignin/PVA nanofibers show smaller fiber diameter, which increases with increasing lignin concentration [60]. A significant reduction in fiber diameter could be due to the removal of non-carbon atoms from lignin during carbonization. As reported by Rangana A. et al. [68], morphological analysis of as-spun nanofibers lignin/PVA nanofibers (dissolved in deionized water) indicated that an increase in PVA concentration increases the average fiber diameter. After carbonization, the lowest fiber diameter was obtained for lignin/PVA with the highest PVA concentration. The viscosity of the precursor solution was determined by PVA, therefore increasing the concentration of PVA led to the formation of thicker nanofibers. However, PVA decomposed during the carbonization of the as-spun nanofibers which resulted in thinner carbonized nanofibers. Similarly, at higher PVA concentration, the viscosity of the solution was high which led to thicker fibers with smaller pore volumes. Apart from facilitating electrospinning of lignin, PVA can be electrospun using nontoxic organic solvents such as DMSO. Therefore, lignin/PVA blend is considered a green approach to electrospinning lignin-based CNF. 

**Figure 11:**
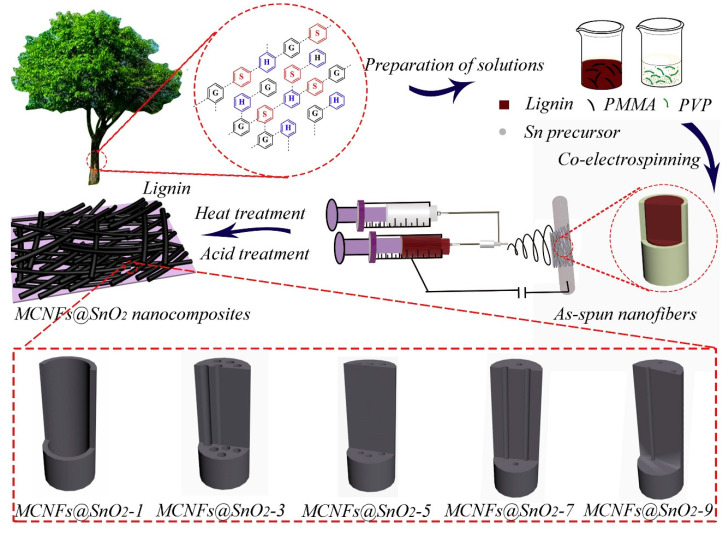
Schematic illustration of the fabrication steps of MCNFs@SnO_2_ nanocomposites at different lignin/PMMA concentration. Reprinted with permission from ref [66].

Poly (methyl methacrylate) (PMMA): Using coaxial electrospinning, M Cao et al. [66] prepared lignin/PMMA as a core electrospinning solution. Composite nanofibers were formed by a partial combination of the core materials with PVP/SnCl_2_ shell material. A schematic illustration of the fabrication of the composite electrode materials is shown in Figure 11. As illustrated in the schematic, composites with different internal structures were formed at different lignin/PMMA ratio in the core solution. At a very low concentration of lignin in (1:9), nearly hollow nanofibers are obtained. This could be due to the complete pyrolysis of PMMA during carbonization. As the mass ratio of lignin increases, the nanofiber becomes denser due to filling up of the core. The hierarchical core surface obtained at maximum lignin mass ratio (9:1), could be due to the inability to electrospin pure lignin nanofibers. In general, PMMA acts as a spinning agent and a sacrificial polymer to increase nanofiber porosity.

Polyvinylpyrrolidone (PVP): Electrospun lignin/PVP and lignin/PVP/Mg(NO_3_)_2_·6H_2_O nanofibers were reported [14]. Improved physiochemical and electrochemical properties of the electrospun nanofibers were due to the presence of Mg(NO_3_)_2_·6H_2_O in the precursor solution. Therefore, PVP only acted as a sacrificial polymer to ease the spinning of lignin into nanofiber and to increase its porosity during carbonization.

Poly(ethylene oxide) (PEO): Using lignin/PEO/Fe(acac)_3_ as shell electrospinning material showed that the properties and performance of the prepared nanofibers are mostly influenced by the concentration of Fe(acac)_3_ in the precursor solution [50]. However, PEO promotes the spinnability and thermal stabilization of lignin nanofibers. Similarly, nanofibers electrospun from a blend of lignin/PEO show fused fibrous morphology with enhanced electrical performance [48]. The controlled degree of fiber fusion could be attributed to beneficial oxygen atoms from PEO. Interconnected nanofibers improve electrical properties by promoting rapid migration of electrolyte ions. However, excessive fiber fusion could result in reduced surface area which can have an adverse effect on the specific capacitance of resultant nanofibers. 

Inorganic additives: Inorganic compounds can be added into lignin precursor solution to modify lignin structure, enabling spinnability and improving quality and performance of resultant nanofiber. For instance, SiO_2_ improved interface miscibility of double capillary and tailored porosity [31], MnO_2_ decorated lignin nanofiber surface with nanowhiskers improved electrochemical performance of lignin-based carbon nanofiber [65], NiCo_2_O_4_ formed a high-performance hybrid supercapacitor with lignin [61], Mg(NO_3_)_2_·6H_2_O produced hierarchical porous lignin-based nanofibers with very high specific capacitance [14] and Fe(acac)_3_ prepared lignin-based hollow carbon nanofibers with superior electrochemical performance [50]. Improved performance due to inorganic additive could be attributed to the pseudocapacitive behavior of those additives.

Influence of electrospinning processing conditions on the morphological properties of electrospun nanofibers.

The morphology and physical properties of electrospun nanofibers depend on the properties of the precursor solution the electrospinning parameter set up. This section discusses the most influential electrospinning on nanofibers.

Single spinneret setup: In the conventional electrospinning setup, a single spinneret comprising a simple hollow needle is used. In the single this configuration, nanofibers are produced by a low flow rate of the precursor solution and the thickness of the fibers are determined by the diameter of the electrospinning needle [74]. To improve the productivity of the single spinneret, an additional electrode can be introduced to increase the number of jets. Tunnapat and Surawut [58] prepared lignin nanofiber using the single spinneret setup by adding glycerol into the spinning solution. Although glycerol improved the spinnability of lignin by reducing the surface tension of the precursor solution, the production of solid (non-hollow) nanofibers of about 21 µm average fiber diameter is attributed to the single spinneret used. By controlling other electrospinning parameters, solid nanofibers of smaller diameters could be produced. For instance, lignin/PVA nanofibers with an average fiber diameter of about 100 nm were prepared using a single spinneret setup [60]. Here, the distance between the spinneret and the needle was the electrospinning parameter responsible for smaller fiber diameter because the distance between the current collector and the spinneret was far enough to allow for maximum stretching of the jet.

Coaxial spinneret setup: The coaxial configuration is used to produce hollow or core–shell structured nanofibers. The coaxial needles are of various diameters and can simply be constructed by inserting one needle into the other [75]. Using coaxial electrospinning setup, F. Mateos et al. [12] prepared lignin and lignin/H_3_PO_4_ as core and shell materials respectively. Such configuration was able to prepare a lignin fiber inside lignin/ H_3_PO_4_ fiber with an average fiber diameter of 1–4 µm. Similarly, a carbon and metal oxide composite nanofiber was prepared from lignin/PMMA and lignin/PMMA/PVP/SnO_2_ as core and shell materials, respectively [66]. The fiber diameter (125–200 nm) of the deposited nanofiber was influenced by other spinning parameters and the carbonization process. Lignin-based hollow CNF fibers were reported by B. Yu et al. [50]. Hollow nanofibers were prepared by evaporation of SAN core solution during the carbonization process of the spun nanofibers. 

Concentration (or viscosity) of precursor solution: For different electrospinning conditions, parameters influence fiber morphology differently. However, the concentration of the precursor solution has been reported to have a major influence on fiber diameter [74,76,77]. At higher concentrations, the viscosity of the electrospinning solution is high which results in larger fiber diameter. Similarly, a very low polymer concentration results in low viscosity which causes the jet to break easily, leading to the formation of beaded fibers. In their article, H. Lee et al. [28] observed randomly distributed fibers with smooth surfaces by dissolving 19 wt % of CA in DMF/acetone (4:6). At 17 wt % CA, beads were observed here and there across fiber distribution. This was an indication that the concentration of CA significantly influenced the formation of beads-free nanofibers. At PVA/PAA ratio of 1/1, ultrafine nanofibers with smallest diameter of 192 nm and narrowest diameter distributions were observed. This could be attributed to the decomposition of non-carbon atoms during carbonization. In another report, CA dissolved in acetone/dimethyl sulfoxide (DMSO) at different concentrations (acetone/DMSO = 4/1, 2/1, 1/1, and 1/2) showed beads-free nanofibers were obtained at acetone/DMSO concentrations of 2/1 [49]. Beads formed at higher and lower concentrations can be due to high and low viscosity, respectively. Therefore, considering a solution that is neither highly concentrated nor highly diluted for electrospinning is very important.

Applied voltage: The effect of applied voltage depends on the interplay between electrostatic force and surface tension. Nanofibers are formed during electrospinning only when there is enough electric force to just overcome the surface tension of the polymer at the nozzle tip. However, higher voltage results in thicker fibers while very low voltage form droplets [78]. X. Wang et al. [27] reported the formation of nanofibers with beads on string by electrospinning CA doped with SiO_2_ at 9 kV. The formation of beads could be attributed to a low voltage which could not sufficiently stretch the jet during spinning. Also, at low voltage, the electric field is not strong enough to completely ionize the polymer solution, letting some of the solvents be trapped in form of beads. In another study reported by F. Mateos et al. [12], lignin/H_3_PO_4_ fiber with an average fiber diameter of 1–4 µm was prepared at spinning voltage of 40 kV. Thicker nanofibers produced could be attributed to high voltage because higher voltage mostly results in thicker fiber diameter.

Flow rate: At a higher flow rate, the solvents do not have sufficient time to evaporate before reaching the current collector. This results in the trapping of the residual solvent on the current collector to form beads. Also, a high flow rate affects ambient condition of the electrospinning environment which could have an adverse effect on fiber morphology [49]. In a study, M. Nasir et al. [30] reported smooth nanofibers prepared by electrospinning 17 wt % CA in DMF/acetone at 0.004 mL/h. Although the concentration of CA in the precursor solution was low, smooth fibers could be attributed to a low flow rate which provided sufficient time for all solvents to evaporate before the jet reaches the collector plate. X. Wang et al. [62] also reported lignin/PAN nanofiber with beaded morphology. Although the beaded structure is mainly due to lignin content in the precursor solution, the high flow rate could also have influenced beads formation.

Distance between nozzle and collector: The effect of spinning distance varies depending on the concentration of the precursor solution and applied voltage. However, too long or too short spinning distance has been seen in many reports to affect the quality of the prepared nanofiber. When the distance between the spinneret and the current collector is too close, beads are formed due to partial evaporation of the solvent [79]. Similarly, at a larger distance, few fibers with random alignment are observed. The reason being that most of the fiber jets do not converge together around the same point on the current collector.

The energy and power density of a supercapacitor is shown in Equations (1) and (2) [5,54].

(1) $$E_{d}=\frac{1}{2}C_{s}V_{sc}^{2}\;({\mathrm{WhKg}}^{-1})$$

(2) $$P_{d}=I_{sc}V_{sc}\;({\mathrm{Wkg}}^{-1})$$

where $E_{d}$, $C_{s},\;V_{sc}$, Q, $P_{d}$, and $I_{sc}$ represent the energy density, specific capacitance, supercapacitor’s cell voltage, electrical charge stored by the supercapacitor, power density and cell current density respectively. Equations (1) and (2) indicate that both the energy density and the power density depend on the cell voltage, which is why an increase in the cell voltage will significantly increase the supercapacitor’s energy and power densities. However, the specific capacitance and the cell voltage of a supercapacitor are strongly dependent on the type of electrode material used by the electrode-electrolyte system [5,54]. Table 5 depicts the performance lignin-based supercapacitors reported in this study.

C. Lai et al. [60] fabricated a supercapacitor based on freestanding lignin/PAN electrode. The fabricated supercapacitor exhibits an energy density of 6 Whkg^−1^ and capacitance retention of 90% after 6000 cycles at 2 Ag^−1^. Alternatively, X. Ma et al. [65] reported a better performing supercapacitor by decorating the surface of lignin/PAN with MnO_2_. The fabricated supercapacitor exhibits a specific capacitance of 84 Whkg^−1^ and capacitance retention of 92% after 10,000 cycles at 2 Ag^−1^. An appreciable increase in performance was due to the pseudocapacitive behavior of MnO_2_. Lignin-based electrode can equally be prepared by coating the active material on a conducting substrate. In such cases, a binder is required to bind the active material to the substrate. Z. Dai et al. [71] reported a supercapacitor based on substrate supported electrode. The electrode slurry was prepared by mixing the active material, carbon black, and PTFE in ethanol. The resultant supercapacitor has an energy density of 18 Whkg^−1^ and capacitance retention of 94.5% after 5000 cycles.

#### 3.2.3. Studies on Electrospun Lignin/Cellulose Nanofibers

The fascinating characteristics of lignin and cellulose nanofibers such as low cost, high carbon performance, wide-field, mechanical stability, and environmental sustainability make them promising candidates for carbon-based EDLC electrode materials. Lignin and cellulose nanofibers can be combined into lignin/cellulose nanofibers to optimize the advantages of lignin and cellulose biomass materials as energy storage devices [54]. Physical blending of lignin and cellulose generally involves modifying the -OH groups phenolic group of lignin and cellulosic material (acetyl group in the case of cellulose acetate) to allow crosslinking reaction between lignin and cellulose macromolecules [36]. To the best of our knowledge, only eight studies on carbon nanofibers derived from the physical blending of electrospun lignin and cellulose precursors were reported.

##### Lignin/Cellulose Acetate (CA) Blend 

**Figure 12:**
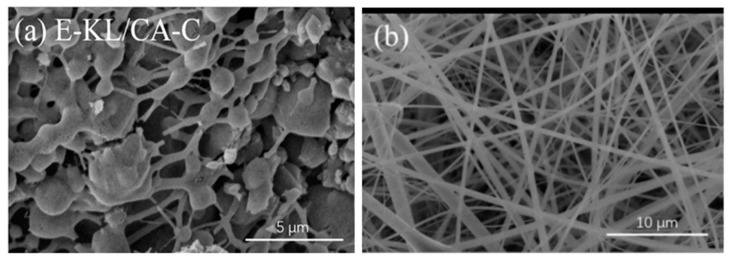
SEM images of electrospun lignin cellulose acetate nanofibers (**a**) after carbonization and (**b**) before carbonization. Adapted with permission from ref [81].

Lignin/CA nanofibers were obtained from continuous dehydration of phenolic hydroxyl group of lignin and cellulosic hydroxyl group to create electron-deficient oxygen atoms. These oxygen atoms can successfully combine with electron-rich atoms from binding agents to form stable bonds between lignin and cellulose acetate [53]. Early attempts to prepare lignin/cellulose fibers met with little success due to the numerous formations of beaded fibers and subsequent collapse of fiber morphology. In 1997, Dave and Glasser reported that adding lignin to cellulose acetate butyrate disrupted the nanocrystalline order of the cellulosic phase [80]. Similarly, electrospun lignin/CA nanofibers with smooth and defect-free morphology were reported [81]. However, fibrous morphologies of these nanofibers were completely lost after carbonization due to phase separation between lignin and cellulose macromolecules. The SEM images of the prepared nanofibers after and before carbonization are shown in Figure 12. Loss of fibrous morphology after carbonization was obviously due to weak interaction between lignin phenolic group and cellulose acetyl group resulting in phase separation.

In an attempt to avoid the morphological collapse of lignin/cellulose nanofibers, M. Schreiber et al. [82] treated electrospun lignin/CA nanofibers with iodine (by vaporization of iodine in a glass jar containing lignin/CA nanofibers followed by recrystallization of iodine vapor) prior to thermal treatment. Iodine treated lignin/cellulose nanofibers were converted to carbon nanofibers (up to 1000 °C). However, the morphological collapse was observed at high CA concentration and at short iodination time. To explain the first scenario, we will believe that the highly aromatic lignin structure readily absorbed iodine which increased the molecular weight of the lignin, thereby improving the formation of nanofibers. On the other hand, CA does not readily absorb iodine due to its nonaromatic structure, thus resulting in two distinct phases in the resultant nanofibers. For the second scenario, iodine uptake by lignin at short iodination time was not sufficient enough to provide stable interaction between lignin and CA, thus leading to morphological collapse during carbonization. 

By adding phosphoric acid (H_3_PO_4_) into lignin/CA precursor solution, stable phospholipid bonds were reported to blend poplar lignin and cellulose acetate [9]. Prepared nanofibers from this blend exhibited the flexibility of CA and the thermal stability of lignin. Beads free fiber morphology at increasing H_3_PO_4_ concentration could be attributed to successful dehydration of hydroxyl group from both lignin and CA by H_3_PO_4_ leading to interphase miscibility. In another paper, epichlorohydrin (ECH) was added to lignin/CA precursor solution to prepare electrospun lignin/CA nanofibers [15]. Presumably, ECH contains epoxy groups which could have reacted with -OH group of lignin to form an epoxy modified lignin. During thermal stabilization, the epoxy group of epoxy modified lignin broke open and reacted with -OH group of CA to form stable covalent bonds between lignin and CA. Nanofibers prepared from covalently blended lignin and CA (using ECH) showed bead-free fibers with complete filamentous morphology at increasing ECH content. Filamentous and beads free nanofiber could mean that stable ECH influenced covalent bond formation between lignin and CA had successfully eliminated phase separation between lignin and CA molecular phases. A much similar approach to electrospinning covalently bonded lignin and CA into fine nanofibers was reported by Z. Dai et al. [83]. In their report, isophorone diisocyanate (IPDI) was used to blend lignin and cellulose acetate. The reaction mechanism for this reaction involves interaction between the isocyanate group of IPDI and hydroxyl group lignin and CA. Good fiber morphology with no evidence of phase separation was due to continuous dehydration of lignin and cellulose hydroxyl group by isocyanate group of IDPI leading to the formation of stable covalent bonds.

##### Lignin/Nanocellulose (NC) Blend

**Figure 13:**
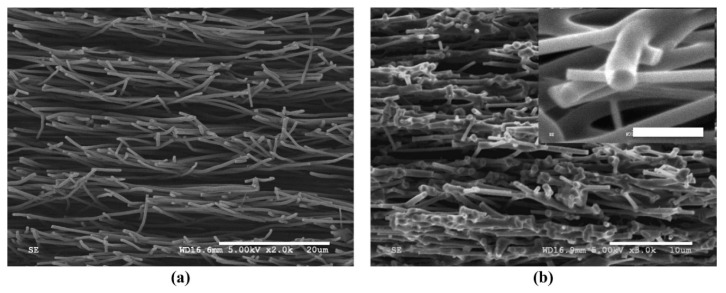
SEM images (cross-sectional view) of lignin nanofiber containing 5 wt % cellulose nanocrystals (**a**) after thermal stabilization and (**b**) after carbonization showing broken fiber structures (magnification 20 µm and 10 µm, respectively). Reprinted with permission from ref [48].

Nanocellulose (also called nanoscale cellulose) is more defect-free, has greater surface area, more active functional groups, and enhanced chemical modification facility than conventional cellulose [84]. Based on their sources, preparation methods, and fiber morphologies, NC can be divided into three categories: (1) cellulose nanocrystals, (2) cellulose nanofibrils, and (3) bacterial cellulose [85]. Pure electrospun lignin/NC nanofibers have not been reported yet. However, the effect of NC on electrospun lignin (blended with other polymers like PVA, PEO, and PAN) nanofibers has been reported recently. M. Ago investigated the effect of cellulose nanocrystals on interfacial properties of electrospun lignin/PVA nanofibers [86,87]. They reported that the addition of cellulose nanocrystals reduced the size of phase-separated domains. This could be because of the molecular mobility of lignin-PVA matrix. Therefore, we can assume that the addition of cellulose nanocrystal could have lowered the dispersion of polymer below the evaporation rate of the solvent thereby reducing phase separation in the nanofibers. In another article, lignin/cellulose nanocrystal/PEO nanofibers were prepared via electrospinning technique [48]. Cellulose nanocrystals were reported to increase the degree of fiber geometry retention after carbonization. The reason for this might be because cellulose nanocrystals prevented lignin from decomposing during carbonization by serving as physical crosslinks for the structure. By skipping the thermal stabilization step, interconnected broken fiber structures were obtained along with all directions of the carbonized nanofibers as shown in Figure 13. This could mean that inclusion of cellulose nanocrystals controlled the degree of interaction between nanofibers.

Hollow nanofibers from electrospun acetylated cellulose nanofibril/silicone oil/chloroform and lignin/PAN as core and shell materials, respectively, were reported [88]. Although not much information on the effect of cellulose nanocrystal on the overall performance of the prepared nanofiber, acetylated cellulose nanofibril-based nanofiber indicates the emergence of an interesting research area for lignin/cellulose-based carbon nanofibers. 

##### Components of Lignin/Cellulose-Based Supercapacitors

An EDLC comprises of four basic components: (1) porous electrode whose active material determines the efficiency of the EDLC, (2) electrolytes which fill the spaces between the electrodes so that ions move through them, (3) ion-permeable separator that allows charged ions from an electrolyte to be moved but not from electrodes, and (4) current collectors that function as conductive devices, capturing and distributing the currents from active materials to the external circuit [89,90,91,92]. Electrospun lignin/lignin nanofibers are carbonaceous electrode materials with highly stable cycle stability, thanks to their high porosity and large surface area. The electrodes can be prepared as freestanding or as substrate-dependent. R. Jayawickramage and P. John [68] fabricated a freestanding activated carbon network electrode based on electrospun PAN blended lignin nanofibers. After activation, the nanofiber mat was used directly as electrode without binder or conductive additive. This supercapacitor recorded a high capacitance of 87 Fg^−1^ and a high energy density of 37 Whkg^−1^. Alternatively, a homogenous slurry containing lignin/CA (active material), carbon black (conductive additive), and PTFE (binder) were coated onto nickel foam (current collector) to form the electrode [9,15,83]. 

##### Electrochemical Performance of Electrospun Lignin/Cellulose Nanofibers

According to literature, electrochemical performance analysis using cycle voltammetry (CV), electrochemical impedance spectroscopy (EIS), and galvanostatic charge–discharge (GCD) was used to study nanofiber energy storage mechanisms. Table 6 presents the electrochemical performance of electrospun lignin/cellulose nanofibers with other carbon-based electrode materials.

All lignin/CA-based carbon nanofibers exhibit high specific capacitance in aqueous electrolytes. This could be attributed to effective cross-linkage between lignin and cellulose leading to effective transport of electrolyte ions. Since the excellent properties of lignin/cellulose acetate nanofibers are attributed to the stable interaction between the phenolic hydroxyl group of lignin and acetyl group of CA, we can compare the performance of lignin/CA nanofibers prepared using different crosslinkers. For an accurate comparison, Table 7 compares the electrochemical performance of the various lignin/CA electrode materials under the same operating conditions.

The energy density of a supercapacitor improves as the average surface area improve [54]. Thus, high energy densities exhibited by lignin/CA electrode materials were due to their large surface area and high specific capacitance. Expectedly, lignin/CA linked by H_3_PO_4_ should have the highest energy density since it has the highest surface area. Contrarily, lignin/CA/ECH recorded the highest energy density despite having the lowest surface area. Lower energy density in the other nanofibers could be attributed to excessive tiny pores which were inaccessible by electrolyte ions. Furthermore, lignin/CA/ECH exhibits the highest specific capacitance which could possibly be due to suitable pore volumes that allow for easy passage of electrolyte ions. Therefore, high energy density in lignin/CA/ECH could be due to high specific capacitance, since the energy density of supercapacitor is proportional to the specific capacitance. The phosphated lignin/CA exhibit the highest carbon content (39.7%) after thermal treatment. This could be attributed to continuous dehydration of phenolic groups in lignin to form stable phospholipid bonds between lignin and CA during phosphoric acid treatment, thus resulting in better thermodynamic stability. Similarly, IPDI cross-linked lignin/CA exhibit the lowest carbon yield because excessive covalent bonds could increase the chances of molecular branched chains breaking, thus decreasing the molecular thermodynamic stability. 

#### 3.2.4. Comparison of Electrospun Cellulose, Lignin, and Lignin/Cellulose Nanofibers

The natural abundance and inherent mechanical flexibility of cellulose make electrospun cellulose nanofiber a fascinating material for flexible supercapacitor. However, the morphological collapse of electrospun cellulose nanofibers during carbonization process remains a serious challenge. Thus, the energy density of cellulose nanofibers is limited by the melting of the nanofibers during carbonization due to the oxidation weightlessness of cellulose at high temperatures [25]. To produce stable cellulose nanofibers, the deacetylation of CA to cellulose has been reported to be an effective strategy. Control of beads formation on the surface of the nanofibers is determined by the hydrolysis time of cellulose acetate precursor. For instance, beads free nanofibers with a glassy structure were obtained by NaOH/ethanol hydrolysis of CA solution at 25 °C for 24 h [26]. Bead-free morphology could be attributed to sufficient hydrolysis time to convert CA to cellulose before thermal treatment. However, the morphological collapse of carbon nanofibers prepared from cellulose precursors is still observed at high and prolonged carbonization temperatures [26,44,101]. 

Due to the low molecular weight and entangled structure of lignin, it is difficult to produce fine nanofibers from lignin by electrospinning technique. To produce electrospun lignin nanofibers, lignin has to be blended with some additives that can modify the structural units of lignin [57,60,69]. Electrospun lignin-based nanofibers exhibit excellent mechanical stability due to the inherent durability of lignin. Cellulose is among the different materials blended with lignin to improve the spinnability of lignin nanofibers. However, electrospun lignin/cellulose nanofibers are not very successful due to morphological collapse of the resultant nanofibers due to phase separation. Phase separation between lignin and cellulose molecular phases are due to weak intermolecular interaction between lignin phenolic groups and cellulose hydroxyl groups [81,82,83]. Observed phase-separated nanofibers show two distinct phases: thermally stable but rigid lignin phase and mechanically flexible but thermally unstable cellulosic phase. SEM images of such nanofibers show numerous beads (indicating phase separation) formed across the surface of the nanofibers. 

Obtaining a nanofiber stable lignin/cellulose nanofiber will is a great achievement in biomass-based supercapacitor not only because of natural abundance and renewable nature of lignin and cellulose, but also because the resultant nanofiber will have the combined properties of lignin and cellulose materials. By mimicking natural interactions of lignin and cellulose in trees, Q. Cao et al. [15], reported lignin/CA acetate nanofibers crosslinked together using covalent bonds. Using ECH as a covalent bond source, stable and beads free CNF from lignin/CA were synthesized. Similarly, phospholipid bonds (via H_3_PO_4_) were used to prepare CNFs from lignin/CA precursor [9]. Interestingly, prepared nanofibers not only retain their morphological properties after carbonization but also exhibit the thermal stability of lignin and mechanical flexibility of CA. Thus, lignin/cellulose nanofiber is by far a better electrode active material compared to individual lignin or cellulose nanofiber.

### 3.3. Motivations

The use of lignin/cellulose nanofibers as electrode materials is innovative for electrochemical double-layer capacitors. This section outlined a few motivations behind using lignin and cellulose nanofibers over other carbon-based nanofibers as supercapacitors electrode materials.

Both lignin and cellulose are biomacromolecules that are found abundantly in nature. The extraction processes of these materials are equally simple and less energy consuming. Thus, lignin/cellulose nanofibers are economically favorable and readily available precursor materials for making carbon nanofiber [78,102,103]. The market for low-cost green and sustainable carbonaceous electrode materials makes lignin/cellulose nanofiber a safer electrode material compared to other costly and harmful materials such as PAN [74,104]. Furthermore, lignin/cellulose nanofibers exhibit the thermal stability of lignin and flexibility of cellulose. The resultant carbon nanofiber maintains its fibrous morphology after carbonization [9,15,83]. Therefore lignin/cellulose nanofiber is a promising electrode material for supercapacitor applications. Biomass-derived CNF surfaces can be doped with metal/semi-metal oxides like Fe_2_O_3_/SiO_2_ to produce defective pores. Supercapacitor electrodes from extremely porous surface materials improve energy storage by providing electrolytic ion pathways that increase the working active volume. Besides, CNF doping increases electric conductivity by supplying electrons for active delocalization of pi-electrons [32,70,105].

The addition of cellulose nanocrystals into lignin/polymer precursor solution reduced phase separation and improved the degree of fiber geometry retention after carbonization [48,86,87]. Composite materials comprising of lignin, CA (or NC), and other polymers (such as PAN, PVA, and PEO) also exhibit outstanding electrochemical performance. Hence, these advances indicate a new research direction on electrospun lignin/cellulose nanofibers for EDLC application [86,87,88]. Although thermal stabilization is extremely important in the fabrication of stable carbon nanofibers, adding cellulose nanocrystals to lignin/cellulose precursor solution controlled the degree of interaction between nanofibers even when the thermal stabilization stage was skipped [48]. 

### 3.4. Challenges

Despite the various advantages of lignin/cellulose nanofiber electrode materials in the field of energy storage, it is difficult to completely embrace these materials due to some of their major limitations. This section will identify the numerous obstacles to the large-scale application of lignin/cellulose-based electrode materials.

While lignin and cellulose are readily available in nature, the use of lignin and cellulose nanofibers in energy storage remains a laboratory application. This, among other factors, can be due to the carbon yield of both lignin and cellulose nanofibers after thermal treatment. After the carbonization process, carbon yield of lignin and cellulose fall as low as 40% and 10–30%, respectively [106]. Poor electrochemical performance is another serious challenge affecting the large-scale application of electrospun lignin/cellulose nanofibers in supercapacitors. Compared to PAN and other petroleum-based carbon materials, the performance of lignin/cellulose nanofiber electrodes lags by a high amount [106,107]. 

The electrospinning technique has the ability to produce fine nanofibers with controlled fiber morphology. However, the quality of nanofiber produced is determined by so many variables. The difficulty in optimizing all these parameters and their collective influence on the prepared nanofiber at the same time remains a serious challenge [75]. This is because a lot of experimental runs need to be done to optimize all the parameters collectively. Such experimental runs are time-consuming and not economical. Finally, to fabricate high-performance supercapacitors based on lignin or cellulose precursor, aqueous electrolytes are mostly used. The use of aqueous electrolyte limits the cell voltage of supercapacitors to about 1.0 V in order to avoid rupture of the supercapacitor as a result of hydrogen gas evolution on the negative electrode and oxygen gas evolution on the positive electrode at higher potentials [5]. 

### 3.5. Recommendations

To address the aforementioned challenges and to meet the current demand for portable and flexible supercapacitors, the most important recommendation is to develop a flexible high-performance solid-state supercapacitor from cost-effective and environmentally friendly materials. Although lignin/cellulose nanofibers electrodes show excellent cycle stability and tailored porosity, their energy density is much smaller than those of other carbon-based electrodes. Therefore, there is a need to improve the performance of lignin/cellulose nanofiber electrodes to compete with other carbon-based electrodes like PAN, graphene, and carbon nanotube without compromising power density. This section will provide a reference for other researchers interested in developing biomass-based supercapacitors as an alternative to the conventional petroleum product-based supercapacitors.

Activation is an effective way of improving the performance of electrospun nanofibers. The activation process can be performed via a physical method (using carbon dioxide or steam) or chemical method (acidic or alkali treatment). Defective pores produced after the activation process serve as suitable electrolyte ions transport channels [32,55]. Therefore, activation of carbonized lignin/cellulose nanofiber will be a simple way to improve the energy density of the resultant nanofiber electrode materials. This is because activation improves pore structures of nanofibers. Another way of improving the energy density of nanofibers is by doping with metallic ions or heteroatoms. Nanofibers doped with heteroatoms or metallic ions exhibit highly improved electrical properties due to the pseudocapacitive behavior of the heteroatoms and metallic ions [54,79,104]. Eventually, doping lignin/cellulose nanofibers with metallic ions or heteroatoms will enhance the electrical performance of the nanofiber appreciably.

Generally, carbon nanofibers with optimum performance were obtained at a limited time, energy, and resource consumption. This process involves the use of optimization models like response surface methodology to optimize parameters whose influence could have direct or interactive effects on nanofiber properties. The model uses central composite design (CCD) to construct experimental design and analysis of variance (ANOVA) to establish statistical validation of regression models as well as to study the interaction of input parameters [36] Hence, employing optimization models to optimize lignin/cellulose nanofibers will certainly save energy and resources. Composite electrode materials comprising of lignin/cellulose backbone are another effective way of improving the electrochemical performance of lignin/cellulose nanofiber electrodes. Composite nanofibers exhibit high electrical performance (due to their pseudocapacitance) when used as supercapacitor electrodes [74,108].

Conventional supercapacitors are composed of current collectors, electrodes, electrolytes, separators, binders, and conductive additives. This makes the supercapacitor rigid and cumbersome to meet practical requirements for portable devices. Electrospun activated carbon fiber network (ACFN) exhibits outstanding performance due to its flexibility, large surface area, excellent mechanical flexibility, and particularly good electrical conductivity. Besides, freestanding ACFNs do not require conductive additives and binders when used as supercapacitors [105,109]. Thus, ACFNs derived from lignin/cellulose nanofibers can be a promising electrode material for biomass-based flexible supercapacitors.

## 4. Conclusions

Biomass carbon nanofiber technology is one of the most active research areas in energy storage devices, especially EDLCs. The main objective of this systematic review is to highlight the state of the art research on electrospun lignin and cellulose nanofibers for application in supercapacitors and to provide directions of future research on the area. A rigorous scientific approach was employed to screen the eligibility of included articles and taxonomy of literature was provided from the most relevant articles included in the literature review. Apart from the articles in the taxonomy of literature, nine other articles were also included in our critical review and development of the research framework. These articles did not strictly meet our inclusion criteria, but they contain vital information that helped in developing the research framework. From our included literature, four main categories were discussed: reviews, studies on electrospun lignin nanofibers, cellulose nanofibers, and lignin/cellulose nanofibers. While studies on electrospun lignin and cellulose-based supercapacitors are considered adequate, very limited studies were reported on lignin/cellulose based supercapacitors. However, the reported studies on lignin/cellulose-based supercapacitors indicate a new research direction for biomass-based supercapacitors. From the articles reviewed in this study, researchers stated some major challenges related to large scale application of these special class of electrode materials. In the same vein, recommendations were given to mitigate these challenges. Specifically, comments and suggestions include:

- Electrospun lignin and cellulose nanofiber-based electrodes have a wide surface area, good porosity, mechanical stability, and excellent cycle stability. However, the energy density of these electrode materials is quite low compared to other carbon-based electrode materials like PAN, graphene, etc. Similarly, carbon nanofibers produced from cellulosic precursors undergo morphological collapse during carbonization due to the low thermal stability of cellulose derivatives. Deacetylation of CA prior to thermal treatment improves the thermal stability of CA-based carbon nanofibers.
- Electrospinning lignin into nanofibers is unrealistic due to the hierarchical and entangled structure of lignin. However, lignin can be easily blended with other binders to produce fine lignin-based nanofibers. Different binders influence electrospun lignin nanofiber differently.
- Electrospinning lignin and cellulose precursor solution resulted in nanofiber with phases separated into lignin and cellulose domains. However, incorporating a suitable crosslinker resolves the problem of phase separation. Resultant nanofibers exhibit thermal stability of lignin and flexibility of CA.
- Lignin/cellulose nanofiber, if explored extensively, can serve as ideal electrode materials for next-generation supercapacitors as an alternative to petroleum-based carbon nanofibers

## 5. Future Directions 

Fabricating cheap and environmentally friendly electrode materials with combined properties of lignin and cellulose could be ideal for next-generation electrospun carbon nanofiber-based supercapacitors. Therefore, future studies must focus on the mechanism of interaction between lignin/cellulose material and other materials at a microscopic level. Similarly, researchers in this area need to adopt combinations of various techniques to improve the overall performance of supercapacitors derived from lignin/cellulose precursors. In effect, the following points will help researches in this area with research directions for future studies:

- Statistical software like response surface methodology (RSM) and intelligent algorithms like artificial neural network (ANN) have been employed to optimize electrospinning parameters of different precursor materials. However, too many experimental runs are required to consider all electrospinning parameters at the same time. Developing optimization software that will consider all electrospinning parameters from a few experimental runs will be revolutionary, not for electrospinning lignin/cellulose nanofiber alone but for all types of fibers.
- The uniqueness of electrospun lignin/CA nanofibers is the formation of high-quality nanofibers with outstanding properties of lignin and CA combined. This property is determined to a large extent by the effectiveness of the linking unit between lignin and CA. Investigating novel crosslinkers that could ensure stable and effective crosslinking reaction as well as improve the performance of lignin/cellulose nanofiber greatly is an exotic area to explore. Interestingly, authors are currently working on this.
- To further improve the performance of electrospun lignin/cellulose nanofibers, researchers should focus on preparing core–shell and hollow nanofibers. This will improve the surface area of the prepared nanofiber as well as combine the performance of different precursor materials used. Employing other techniques—such as hydrothermal, template synthesis, in situ polymerization, etc.—to augment electrospinning technique is also a wonderful research direction for fabricating lignin/cellulose-based supercapacitors.
- A hybrid supercapacitor benefits from the performance of an EDLC (high energy density) and pseudocapacitors (high power density). Due to high surface area, high porosity, mechanical and thermal stability, excellent cycle life, and sustainable nature of lignin/cellulose nanofibers, electrospun lignin/cellulose based asymmetric supercapacitors would be an exotic research area. Researchers should focus on asymmetric supercapacitors comprising of lignin/cellulose nanofiber as an EDLC electrode and conducting polymer, MOF, or metal oxide as a pseudocapacitive electrode. Authors are also working on this.
- The development of flexible solid-state supercapacitors is taking a new dimension with the recent demand for flexible electronic devices. To meet this demand, fabricating an all-solid-state lignin/cellulose-based supercapacitor is an interesting area to explore because of its mechanical flexibility and high performance. In our next article, we will report the performance of all-solid-state supercapacitor derived from lignin/cellulose nanofiber and PVA-based electrolyte.
- The efficiency of flexible supercapacitors decreases significantly due to the distortion in the configuration of the supercapacitor after many bending cycles. Researchers need to concentrate on incorporating self-healing materials into lignin/cellulose nanofibers to allow the supercapacitor to heal itself following persistent bending cycles.

## Figures and Tables

**Table 1:** Details of query search for relevant articles from 2010 to 2020

Step	Keyword Search	Number of Documents			
		Web of Science (All Databases)	Scopus	Science Direct	Total
1	Electrospinning [Title]	6364	6977	24,920	38,261
2	Electropun [Title]	12,372	12,927	19,608	44,907
3	Nanofiber [Title]	8178	23,502	50,126	81,806
4	Supercapacitor [Title]	10,265	12,185	32,400	54,832
5	EDLC [Title]	148	205	8096	8449
6	Cellulose [Title]	32,776	34,311	192,250	259,337
7	Lignin [Title]	11,088	11,684	66,056	88,828
8	Step 1 + 3 + 6	17	69	4350	4436
9	Step 1 + 3 + 7	0	2	685	687
10	Step 2 + 3 + 6	41	172	3925	4138
11	Step 2 + 3 + 7	11	30	642	683
12	Step 1 + 3 + 4 + 6	0	0	487	487
13	Step 1 + 3 + 4 + 7	0	0	150	150
14	Step 1 + 3 + 5 + 6	0	0	134	134
15	Step 1 + 3 + 5 + 7	0	0	46	46
16	Step 2 + 3 + 4 + 6	4	9	408	421
S	Step 2 + 3 + 4 + 7	1	4	134	139
18	Step 2 + 3 + 5 + 6	1	2	91	94
19	Step 2 + 3 + 5 + 7	0	1	34	35
20	Step 8–19	75	289	11,086	11,450

**Table 2:** Inclusion criteria for selected articles

Electronic databases	Web of Science (http://apps.webofknowledge.com/) Scopus (https://www.scopus.com/) ScienceDirect (https://www.sciencedirect.com/)
Other sources of information	ResearchGate (https://www.researchgate.net/) Researcher (https://www.researcher-app.com/)
Search items	Journal papers, review articles, conference proceedings, and book chapters
Publishing language	English
Publication period	2010–2020

**Table 3:** Properties of EDLC electrode materials depending on their methods of fabrication and the optimization of parameters.

Method	Precursor	Remark	Shortcomings	Ref.
Chemical Vapor Deposition	Lithium titanite	Excellent electrical conductivity High specific gravity High thermal conductivity	High cost of production Hydrophobic surface	[6]
Electrospinning	Lignin	Easy diameter adjustment Post spinning treatment (carbonization) is possible	Long processing time Low yield of CNFs	[36]
Pyrolysis	Bamboo	Short processing time High carbon nanofiber yield	Difficult to adjust fiber diameter Carbonization is not possible	[37]
In situ polymerization	Polyacrylonitrile solution	Good cycling stability Binder free PmAP/CNF hybrid electrode	Low carbon yield Low specific capacitance	[34]
Electrospinning	Cellulose acetate	High specific capacitance Excellent cycling stability specific energy density	Scalable friction remains a problem	[38]

**Table 4:** Processing conditions for electrospun lignin nanofibers.

Precursor	Electrospinning Parameters	Solvent	Thermal Treatment			Fiber Diameter	Surface Area (m^2^/g)	Ref.
			**Stabilization**	**Carbonization**	**Activation**			
Alkali lignin/glycerol	Needle: Single Voltage: 20 kV Distance: 15 cm	Deionized water	Air (260 °C for 1 h at 1 °C/min)	Nitrogen gas (800 °C for 1 h at 5 °C/min)	-	21.05 ± 9 µm	85.67	[58]
Alcell Lignin/H3 PO4 (shell)	Needle: Coaxial Voltage: 40 kV Flow rate: 0.1 (1) mL/h	Ethanol	Air (200 °C for 1 h at 1 °C/min)	Nitrogen gas (900 °C)	Oxygen gas (0.5, 3.5 and 6.5%)	1–4 µm	2340	[12]
Corn stalk modified lignin/IPDI	Needle: Single Voltage: 20 kV Distance: 18 cm Flow rate: 0.8 mL/h	DMF/acetone	Air (220 °C for 4 h at 0.5 °C/min)	Nitrogen gas (1000 °C for 4 h at 4 °C/min)	KOH (25 ℃ for 12 h)	260 nm	2042.86	[57]
Lignin/PAN/BA	Needle: Single Voltage: 20 kV Distance: 18 cm Flow rate: 5 µL/min	DMF	Air (200 °C for 12 h at 0.2 °C/min)	Argon gas (1000 °C for 30 min at 0.5 °C/min)	-	1690 ± 70 nm	-	[59]
Kraft lignin/PVA	Needle: single Voltage: 26 kV Distance: 25 cm Flow rate: 1.2 L/h	Distilled water	Air (220 °C for 8 h at 0.05 °C/min)	Argon gas (1200 °C for 1 h at 10 °C/min)	-	~100 nm	~583	[60]
Lignin/SiO_2_ (core)	Spinning: Coaxial Voltage: 20 KV Distance: 20 cm Flow rate: 0.08 (0.1) mm/min	DMF/ethanol	-	Nitrogen gas (900 °C for 2 h at 5 °C/min)	-	-	870	[31]
Lignin/PAN/NiCo_2_O_4_	Spinning: Single Voltage: 8.5 KV Distance: 20 cm	DMF	Air (280 °C for 1 h at 1 °C/min)	Nitrogen gas (1000 °C at 5 °C/min)	-	~270 ± 15 nm		[61]
Lignin/PAN	Spinning: Single Voltage: 25 KV Distance: 25 cm Flow rate: 4.8 mL/min	DMF	Air (250 °C for 90 min at 1 °C/min)	Nitrogen gas (800 °C for 60 min at 10 °C/min)	-	172 nm	675	[62]
Lignin-g-PAN/MnO_2_	Spinning: Single Voltage: 12 KV Distance: 15 cm Flow rate: 10 μL/min	DMF	Air (250 °C for 2 h at 10 °C/min)	Nitrogen gas (1400 °C for 30 min at 10 °C/min)	-	400–600 nm		[63]
Lignin/PEO/Fe_3_ O_4_ (shell)	Spinning: Coaxial Voltage: 25–30 kV Distance: 15–20 cm Flow rate: 1 (0.4) and 1 mL/min	DMF	Air (250 °C for 1 h at 0.2 °C/min)	Nitrogen gas (900 °C for 1 h at 0.3 °C/min)	-	~400–600 nm	281	[50]
Lignin/PAN	Spinning: Single Voltage: 15 kV Distance: 15 cm Flow rate: 0.3 mL/min	DMF	Air (250 °C for 1 h at 5 °C/min)	Argon gas (900 °C for 1 h at 5 °C/min)	-	-	-	[64]
Lignin/PEO	Spinning: Single Voltage: 24–20 kV Distance: 18 cm Flow rate: 1.0–1.1 mL/min	NaNO_3_/NaOH	Air (250 °C at 10 °C/min)	Nitrogen + Hydrogen gas (1400 °C for 30 min)	-	188 ± 38 nm	-	[16]
Lignin/PVA/MnO_2_	Spinning: Single Voltage: 26 kV Distance: 25 cm Flow rate: 1.2 mL/min	Distilled water	Air (220 °C for 8 h)	Argon gas (1200 °C for 60 min)	-	~200 nm	583	[65]
Lignin/PMMA (core)	Spinning: Coaxial Voltage: 24 kV Distance: 25 cm Flow rate: 7.1 (5.3) µL/min	DMF/ethanol	Air (300 °C at 1 °C/min)	Nitrogen gas (800 °C for 2 h)	HF and HNO_3_ at 25 °C for 5 h	125–200 nm	659	[66]
Lignin/PVP/Mg(NO_3_)_2_·6H_2_O	Spinning: Single Voltage: 15 kV Distance: 20 cm Flow rate: 0.2 mL/min	DMF	Air (350 °C for 4 h at 1 °C/min)	Nitrogen gas (800 °C for 1 h at 3 °C/min)	6 mol/L HCl	124 nm	1140	[14]
Lignin/Plant protein	Spinning: Single Voltage: 20 kV Distance: 20 cm Flow rate: 1 mL/h	Acetic acid/DMF	Air (250 °C for 3 h at 0.5 °C/min)	Nitrogen/Argon = 5/95 (900 °C for 2 h at 5 °C/min)	Argon/CO_2_ = 95/5 (850 °C for 3 h)	0.85 ± 0.24 μm	1113.16	[67]
Lignin/PVA	Spinning: Single Voltage: 20 kV Distance: 15 cm Flow rate: 0.8 mL/h	Deionized water	Air (250 °C for 1 h at 2 °C/min)	Helium gas (1000 °C for 1 h at 5 °C/min)	Nitrogen and CO_2_ (800 °C for 30 min at 130 °C/min)	175 ± 25.5 nm	2170	[68]
GN modified lignin/PAN	Spinning: Single Voltage: 15 kV Distance: 15 cm Flow rate: 1 mL/h	DMF	Air (260 °C for 3 h at 0.5 °C/min)	Helium gas (1400 °C for 1 h at 5 °C/min)	KOH (ultrasonication for 1 h)	-	2439	[69]
Lignin/PAN/Pitch/Zn	-	DMF/THF	Air (280 °C for 1 h)	Inert atmosphere (800 °C for 1 h at 5 °C/min)	-	370 nm	1194	[70]
Lignin/PAN	Spinning: Single Voltage: 20 kV Distance: 18 cm Flow rate: 1 mL/h	DMF	Air (200 °C for 1 h at 0.2 °C/min)	Nitrogen gas (1000 °C for 1 h at 5 °C/min)	KOH (dispersion for 1 h) Inert atmosphere (600 °C for 1 h)	411.7 ± 55.9 nm	2313	[71]
Lignin/PAN/PANI/urea	Spinning: Single Voltage: 15 kV Distance: 15 cm Flow rate: 0.5 mL/h	DMF	Air (260 °C for 1 h at 1 °C/min)	Nitrogen gas (800 °C for 2 h at 5 °C/min)	-	150–250 nm	483.1	[72]
Lignin/PEO/NCC	Spinning: Single Voltage: 20 kV Distance: 25 cm	DMF	Chromatography oven (250 °C for 1 h at 5 °C/min)	Nitrogen gas (800, 900 and 1000 °C for 1 h at 1, 5 and 10 °C/min)	-	406 ± 51 nm	-	[48]
Lignin/PAN	Spinning: Single Voltage: 19 kV Distance: 16 cm Flow rate: 0.8 mL/h	DMF	Air (280 °C for 1 h at 2 °C/min)	Nitrogen gas (1000 °C for 1 h at 5 °C/min)	CO_2_ (50 mL/min) once carbonization temp reaches 1000 °C	79.35 ± 8.58 nm	2543	[73]

**Table 5:** Literature summary for physical blending of electrospun lignin nanofibers.

Lignin Type	Physical Blending	Electrode Type	Electrolyte	Specific Capacitance (F/g)	Energy Density (Wh/kg)	Power Density (kW/kg)	Capacitance Retention	Ref.
Kraft lignin	Blending with PVA	Freestanding	6 M KOH	64	5.67	0.94	90% after 6000 cycles at 2 A/g	[60]
Alkali lignin	Blending with PVA + MnO_2_	Freestanding	1.0 M LiPF6	83.3	84.3	5.72	92% after 10,000 cycles at 2 A/g	[65]
Lignin	Blending with PEO	Freestanding	EMIMBF4	133	86.6	114	94% after 10,000 cycles at 4 A/g	[31]
Alkali lignin	Blending with PAN + NiCo_2_O_4_	Freestanding	PVA-KOH	134.3	47.75	0.799	74.22% after 5000 cycle at 50 mA/cm^2^	[61]
Sawdust lignin	Blending with PMMA	Coating	6 M KOH	406	11.5	0.451	95% after 10,000 cycles at 10 A/g	[66]
Alkali lignin	Blending with PVP + Mg(NO_3_)_2_·6H_2_O	Freestanding	6 M KOH	248	-	-	97% after 1000 cycles at 20 A/g	[14]
Enzymatic hydrolysis lignin	Blending with PAN	Freestanding	6 M KOH	305.7	-	-	92% after 1000 cycles at 1 A/g	[62]
Alkali lignin	Blending with PAN	Freestanding	PVA–KOH	129.23	2.63	4.49	92% after 1000 cycles	[64]
Lignin	Blending with plant protein (hordein/zein = 50/50, *w*/*w*)	Coating	6 M KOH	410	-	-	95% after 3000 cycles	[67]
Alkali lignin	Blending with PVA	Freestanding	Pyr14TFSI/PC/EC	88	38	1.666	87% through 1000 cycles	[68]
Methanol soluble lignin (isolated from kraft lignin)	Grafting with PAN + MnO_2_	Freestanding	1 M Na_2_SO_4_	171.6	6	160	99 after 1000 cycles	[63]
Acetic acid lignin	Blending with PEO + Fe(acac)_3_	Coating	1 M Na_2_SO_3_	121			90% after 1000 cycles	[50]
Cornstalk residues lignin	Blending with PAN + GN	Coating	6 M KOH	267.32	9.28	0.493	96.7% after 1000 cycles	[69]
Hardwood lignin	Blending with PAN + Pitch + Zn	Freestanding	6 M KOH	165	22	0.4	88% after 3000 cycles	[70]
Organosolv lignin (butyric anhydride esterified)	Blending with PAN	Coating	6 M KOH	320	17.92	0.8	94.5% after 5000 cycles	[71]
Lignin	Blending with PANI + PANI + urea	Freestanding	1 M H_2_SO_4_	199.5	-	-	88% after 1000 cycles at 4 A/g	[72]
Alkali Kraft lignin	Blending with PAN	Freestanding	Pyr14TFSI:PC:EC	128	59	15	-	[73]

**Table 6:** Electrochemical performance of carbon-based supercapacitors compared

Carbon Materials	Electrode System/Electrolyte Type	Current Density or Scan Rate (A g^−1^)	Specific Capacitance (F g^−1^)	Ref.
PEDOT/Lignin biopolymer	3E/0.1 M HClO_4_	1	170.4	[93]
Corncob residue	3E/6 M KOH	1	120	[94]
Pongam seed shells	3E/1 M KOH	1	94	[95]
Lignin-based hydrogel	1 M KOH	0.5	129.3	[64]
Wood	3E/6 M KOH	0.2	246	[96]
Waste cotton	2E/1 M TEABF_4_/AN	1	87	[97]
Silk	2E/1 M H_2_SO_4_	0.1	264	[98]
Willow catkin	3E/6 M KOH	1	292	[99]
Glucose-derived carbon	3E/1 M H_2_SO_4_	0.2	206	[100]
KL/CA (ECH) CNFs	2E/6 M KOH	0.1	346.6	[15]
KL/CA (H_3_PO_4_) CNFs	2E/6 M KOH	0.1	320.3	[9]
KL/CA (IPDI) CNFs	2E/6 M KOH	0.1	174.62	[83]

**Table 7:** Electrochemical performance of lignin/cellulose-based electrodes using 6M KOH electrolyte

Precursor Material	Cross-Linker	Pore Diameter (nm)	Fiber Diameter (nm)	Specific Capacitance (Fg^−1^)	Energy Density (Whkg^−1^)	Power Density (kWkg^−1^)	Capacitance Retention (% at 5 A g^−1^)	Surface Area (m^2^ g^−1^)	Carbon Yield (%)	Ref.
Lignin/CA	ECH	2.3345	370 ± 24	346.6	31.5	400	77.3	837.4	32	[15]
Lignin/CA	H_3_PO_4_	2.17	340 ± 32	320.3	30.2	400	70.6	1061.7	39.7	[9]
Lignin/CA	IPDI	2.0259	760 ± 32	174.62	6.07	450	90	1013.8	25.45	[83]
